# Treatment of early-stage diabetic nephropathy with Siddha drug Sirupeelai Kudineer: A case series

**DOI:** 10.1016/j.jaim.2024.100993

**Published:** 2024-12-02

**Authors:** P. Parvathy, G.S. Lekha, S. Aparna, A. Kanagarajan

**Affiliations:** Research Associate, Siddha Regional Research Institute, Thiruvananthapuram, India

**Keywords:** Siddha, Diabetic nephropathy, Sirupeelai kudineer, eGFR, Serum creatinine

## Abstract

Diabetic Nephropathy is one of the major microvascular complications of Diabetes Mellitus, which can be detected in the earlier stage by investigating urinary microalbumin excretion levels and estimating ACR and GFR. Early treatment can delay or prevent the progression of diabetic Nephropathy. Monoherbal Siddha formulation Sirupeelai Kudineer indicated for the complications of Diabetes Mellitus is selected for this Case study. The efficiency of the drug was assessed by measuring the change in renal function throughout the treatment period and the KD QOL assessment. Four patients presented with symptoms of Diabetic Nephropathy were treated with the monoherbal Siddha formulation *Sirupeelai Kudineer*. This series of four cases showed improvement in CKD QOL and some biochemical parameters. Blood urea was reduced in the four cases during medication. Serum creatinine levels mildly increased in three cases somewhere during the treatment period but decreased in all four cases after treatment. The estimated GFR also improved in three cases after treatment but mildly reduced in one patient. Symptomatic improvement was also observed in all the patients. Marked reduction in blood urea and serum creatinine levels after treatment shows the Nephroprotective action of the drug. The clinical and laboratory parameters observed among these four patients suggest that this drug may be used along with other hypoglycemic drugs to treat Diabetic Nephropathy.

## 1 Introduction

Diabetic Kidney Disease (DKD), a common microvascular complication of type I and type II diabetes, is characterized by increased albuminuria level or urinary albumin-to-creatinine ratio (UACR), decreased glomerular ﬁltration rate (GFR) or both [1]. Diabetic Nephropathy is a major cause of chronic kidney disease and end-stage renal failure globally [2]. Structural and functional changes occur in the Kidney because of Diabetes and result in proteinuria, hypertension, and progressive reduction of kidney function, which is the hallmark of diabetic Nephropathy [3]. The classical presentation of Diabetic Nephropathy is characterized by hyperfiltration and albuminuria in the early phases, which are then followed by a progressive loss of filtration and declined renal function [4].

Siddha Literature describes the complications of Diabetes (*Neerizhivu noi*) as ten types of *avathaigal* in *Neerizhivu noi* [5]. The features mentioned in avathaigal illustrate the clinical outcome of chronic kidney diseases, such as oliguria, fatigue, anorexia, nausea, vomiting, itching and dryness of skin, drowsiness, numbness, and swelling in the limbs, muscle twitching or cramps, bone pain, breathlessness, increased thirst, sleep disturbance, and sexual problems [5].

Clinical evidence suggests that apart from Dialysis, there is no curative or preventive treatment for Diabetic Nephropathy. However, evidence shows that early treatment can delay or prevent the disorder's progression [6]. Hence, there is a need to find out reliable treatments to slow the progression of diabetic complications like diabetic Nephropathy. In Diabetes, some herbal alternatives provide symptomatic relief and prevent secondary disease complications [7].

The drug, *Sirupeelai* (Aerva lanata), is mentioned as *Pashana bedhi* and *Uppu chatthai nasamakki* in the Siddha literature. It can be taken as an indicator of removing metabolic waste products from blood [8]. The Nephroprotective activity of the herb *Sirupeelai* (Aerva lanata), pharmacologically evaluated in animals, showed that the ethanolic extract of A. lanata has critical potential as a nephroprotective agent [9]. The decoction of the herb *Sirupeelai* (Aerva lanata) has also been evaluated in animal models and showed a remarkable Nephroprotective activity *via* biochemical parameters and histopathological analysis [10]. Studies also show the antioxidant and anti-diabetic activity of the herb *Sirupeelai* [11,12]. The plant extract is possessed to have Diuretic, Nephroprotective, Hepatoprotective, Immunomodulatory, Anti-hyperglycemic, and Anti-inflammatory properties [13].

In Siddha literature, the drug *Sirupeelai* is indicated for *Thrithodam, Neeradaippu*/*Moothira Kiricharam* (Urinary disorders)*, and Pandu* (Anaemia) [14], which are all mostly found as the later-stage complications of Diabetes mellitus. The drug *Sirupeelai* (*Aerva lanata*) is of bitter (*Kaippu*) taste and hot potency (*Veppa veeriyam*) [14]. The *Kaippu* (bitter) taste substances can balance the elevated *Iya* humor and aggravate the *Vali* humor [15]. Hence *Sirupeelai* can neutralize *Iyam* and arouse the *Abana Vayu*, thereby supporting the elimination of deranged metabolic wastes and other impurities from the body. The central action of the herb *Sirupeelai* is Diuretic [14] which is essential for removing stagnant impurities and water from circulation and the functioning of kidneys. Hence the drug *Sirupeelai Kudineer* (Whole plant decoction of Aerva lanata) was selected to treat the patients affected with Diabetic Nephropathy. The decoction form is chosen since it is cost-effective, safe, and relatively easy to prepare. The present article is an observational outcome of cases treated with the monoherbal siddha formulation, *Sirupeelai kudineer*.

## 2 Patient information

### 2.1 First case

A 69 -year-old male afflicted with Diabetes mellitus for 12 years was on insulin for a year. The patient had dyslipidemia for six years and hypertension for a year. He complained of increased frequency of micturition four times during the night, fatigue, shortness of breath on exertion, and numbness in his hands and feet. The patient had no abusive habits like smoking, alcohol intake, or betel nut chewing. He was on Tablet Glycozide 40 half tablet daily, Tablet Contenel 20 daily once, Tablet Dysliptin 5 Once daily, Tablet Prazopill XL 5 once daily, Tablet Hytel 40 twice daily, and Insulin Novomix in the dose of 10 units before breakfast, eight units before dinner subcutaneously. The patient stopped taking Tablet Dysliptin 5 and Tablet Prazopill 5 XL before taking Sirupeelai kudineer since his serum cholesterol level was normal, and his blood pressure also seemed to be under control. Other medicines continued as such. His blood pressure was 140/80 mm Hg, and other vital signs were normal. His physical examination was unremarkable, and respiratory, cardiovascular, and abdominal examinations were normal. Ultra-sonogram (Pelvis and abdomen) was normal. His laboratory examination was significant for change in renal profile (Refer to Table 1).

**Table 1:** Summary of biochemical parameters before, during, and after treatment.

Cases	Before Treatment	Visit I	Visit II	Visit III	Visit IV	After Treatment
**Blood urea (mg/dl)**						
Case I	35.4	38.4	33.2	48.3	36.7	22.6
Case II	20.1	22.5	20.9	19.5	∗	∗
Case III	47.4	38.8	42.5	40.1	31.8	∗
Case IV	31.2	28.8	42.2	∗	∗	∗
**Serum Creatinine (mg/dl)**						
Case I	1.59	1.49	1.59	1.63	1.72	1.14
Case II	1.09	1.28	1.27	1.07	∗	∗
Case III	1.57	1.57	1.57	1.65	1.52	∗
Case IV	1.21	0.99	1.2	∗	∗	∗
**Urine Microalbumin (mg/L)**						
Case I	362.3	71.62	85.82	141.70	263.3	1470.8
Case II	181.36	29.30	52.36	42.33	∗	∗
Case III	157.35	109.64	33.39	112.03	89.91	∗
Case IV	216.45	45.65	53.74	∗	∗	∗
**ACR (mg/mmol)**						
Case I	119.71	43.05	39.58	24.90	107.80	160.49
Case II	54.94	25.08	36.98	24.53	∗	∗
Case III	22.94	24.78	12.45	33.49	19.10	∗
Case IV	19.30	5.36	6.17	∗	∗	∗
**eGFR (ml/min)**						
Case I	34.1	32.7	34.7	37.7	33.6	38.7
Case II	89.4	76.2	75.9	91.1	∗	∗
Case III	47.5	45.2	47.5	43.6	46.7	∗
Case IV	90.9	110	90.7	∗	∗	∗
**Urea Creatinine Ratio**						
Case I	22.3	25.8	20.9	29.6	21.3	19.8
Case II	18.4	17.6	16.4	18.2	∗	∗
Case III	30.1	24.7	27.1	24.3	20.9	∗
Case IV	25.8	29.1	35.2	∗	∗	∗
**Urine Creatinine (mg/dl)**						
Case I	34.2	18.8	24.5	64.3	27.6	136.4
Case II	37.3	13.2	16	19.5	∗	∗
Case III	77.5	50	30.3	37.8	53.2	∗
Case IV	126.7	96.2	98.4	∗	∗	∗
**Blood Sugar Level (mg/dl)**						
Case I	FBS –104.7 PPBS -111.7	RBS – 142.4	RBS – 157.2	RBS – 154	FBS –117 PPBS -250	FBS – 92.7 PPBS – 175.4
Case II	FBS –141.6 PPBS -302	RBS – 320.1	RBS – 162.6	RBS – 250.9	∗	∗
Case III	FBS –171 PPBS -368.5	RBS – 143.6	RBS – 334.2	RBS – 503.1	FBS –210.8 PPBS -378.5	∗
Case IV	FBS – 220 PPBS – 371.8	RBS – 217.4	RBS – 287.4	∗	∗	∗
**Urine routine and microscopic**						
Case I	Sugar – Nil, Protein loss 30 mg/dl, bacteria seen	Sugar – Nil Protein – Nil, bacteria seen	Sugar – Nil, Protein - Nil, No bacteria seen	Sugar – Nil Protein - Nil	Protein loss 30 mg/dl.	Protein loss 30 mg/dl.
Case II	Sugar – Nil, Protein loss 30 mg/dl	Sugar – Nil Protein – Nil	Sugar – 250 mg/dl Protein - Nil	Sugar – Nil Protein - Nil	∗	∗
Case III	Sugar – Nil, Protein loss 30 mg/dl	Sugar – Nil Protein - Trace	Sugar – 250 mg/dl Protein - Nil	Sugar – 500 mg/dl Protein – 30 mg/dl	Sugar – 500 mg/dl Protein - Nil	∗
Case IV	No Protein and Sugar loss in urine.	Sugar – 250 mg/dl Protein - Nil	Sugar – 250 mg/dl Protein - Nil	∗	∗	∗
**Blood Pressure (mm of Hg)**						
Case I	140/80	110/70	110/80	110/90	140/80	130/80
Case II	140/90	140/80	130/90	130/80	∗	∗
Case III	160/80	140/80	140/80	130/70	140/80	∗
Case IV	140/80	130/80	130/70	∗	∗	∗

∗ Patient did not come for review after that.

### 2.2 Second case

A 62-year-old male patient complained of numbness in hands and feet, muscle cramps in calf muscles, especially during the night, and generalized, persistent itchy skin for two years, which was aggravated for one month. His medical history was significant for type 2 diabetes mellitus for the past five years. The patient denied any history of abusive habits like smoking or illicit drug use. His home medications included Metformin hydrochloride 500 mg by mouth twice daily and Glimepiride 1 mg by mouth twice daily. Initially, the blood pressure was 130/90 mmHg, and other vital signs were typical. His physical examination was not significant. The patient was told to stop all other medicines and to take only *Sirupeelai kudineer*. Laboratory testing was significant for change in renal profile (Table 1). Ultra sonogram (Pelvis and abdomen) revealed no abnormality. Other biochemical investigations were within normal limits.

### 2.3 Third case

A 68-year-old male attended the OPD with the chief complaints of swelling in both legs, hyperpigmentation, itching in feet, and often tiredness. The patient reported that his main symptoms were swelling and itching in both legs, which gradually onset. Similar symptoms were recurring on prolonged standing and relief on taking rest. H/o Diabetes mellitus for nine years, and he was treated with ayurvedic medicines. No history of hypertension or dyslipidemia. His blood pressure was 160/80 mmHg, and other vitals were normal. His physical examination and systemic examinations were unremarkable. Ultra-sonogram (Pelvis and abdomen) studies in normal. No family history of Diabetes mellitus, and no other co-morbidities were present. He stopped the ayurvedic medicines before taking *Sirupeelai kudineer*. His laboratory data are summarized in Table 1.

### 2.4 Fourth case

A 60-year-old male patient presented with complaints of bilateral pedal edema, general weakness, and frequent nocturnal micturition for the last six months. The patient's medical history was significant for type 2 diabetes for the past 20 years. The patient was taking injections of Huminsuline 30/70 in the dose of 30 units before lunch and 30 units before dinner subcutaneously. The patient had no hypertension, dyslipidemia, or other chronic disease history. He had no habit of smoking or illicit drug use. The patient was advised to continue insulin injection along with siddha medicines. His blood pressure was 140/80 mm Hg, and other vital signs were also typical. His physical examination was unremarkable; respiratory, cardiovascular, and abdominal examinations also revealed no significant pathological changes. Ultra sonogram (Pelvis and abdomen) showed no abnormality. However, his laboratory examination was substantial for a change in renal profile. The laboratory data of the patient are summarized in Table 1.

## 3 Clinical findings

It is critical to diagnose patients more sensitive to developing Diabetic Nephropathy for better control of the disease process. Therefore, it is significant to use different strategies for earlier detection of Diabetic Nephropathy in Diabetes mellitus helps to slow down the progression of the disease. In addition, early treatment reduces Diabetic Nephropathy prevalence and increases the quality of life among people with diabetes mellitus. For the earlier detection of diabetic Nephropathy, Micro-albumin, GFR, and UACR were estimated. Other baseline investigations were also done. Findings are summarized in Table 1. Ultra sonogram (Pelvis and abdomen) was done to rule out other renal pathologies. Symptoms noted in Siddha assessments and outcomes were listed in Table 2, quality of life of the patient was assessed through the CKD QOL Questionnaire marked in Supplementary Table 2. Kidney function tests are performed once a week during the period of drug administration; findings were summarized in Table 1; symptoms noted, Siddha assessments and outcomes were listed in Table 2; quality of life of the patient was assessed through the CKD QOL Questionnaire marked in Supplementary Table 2. In addition, blood investigation and renal function tests are performed after completing the medication routine (see Supplementary Table 1).

**Table 2:** Siddha assessment - Before Treatment (BT) and After Treatment (AT).

*Envagai thervu* (Eight Methods of Examination)	Case I		Case II		Case III		Case IV	
	BT	AT	BT	AT	BT	AT	BT	AT
*Neerkkuri* (Urine examination)	Straw colored	Straw colored	Yellowish	Yellowish	Straw colored	Straw colored	Straw colored	Straw colored
*Neikkuri* (Oil on urine sign)	Pearl Shaped, not spreading- Kapha neer	Round pattern, Steady spread –Pitha neer	Linear initially, then spread to sides – Vatha neer	Linear initially, then spread all over to sides – Vatha pitha neer	Kidney shaped - Vatha pitha neer	Kidney shaped- Vatha pitha neer	Pearl shaped – Kapha neer	Parrot beak shaped – Kapha vatha neer
*Naadi* (Pulse)	*Pitha Vatham*	*Vatha Pitham*	*Pitha Vatham*	*Pitha Kapham*	*Pitha Vatham*	*Pitha Vatham*	*Pitha Vatham*	*Vatha Pitham*
*Sparisam* (palpation)	Mild warmth present	Normal	Mild warmth present	Mild warmth present	Normal	Normal	Mild warmth present, sweat increased	Mild warmth present, sweat increased
*Naa* (Tongue examination)	Coating, Fissure, Dryness absent	Coating, fissures-nil, taste perception -normal	Coating, fissures-nil, taste perception -normal	Coating, fissures-nil, taste perception -normal	Coating, fissures-nil, taste perception -normal	Coating-present, fissures-nil, taste perception -normal	Coating-present, fissures-nil, taste perception -normal	Coating-present, fissures-nil, taste perception -normal
*Niram* (Colour of the body)	Dusky complexion	Dusky complexion	Wheatish complexion	Wheatish complexion	Wheatish complexion	Wheatish complexion	Dusky complexion	Dusky complexion
*Mozhi* (Speech)	Low pitched	Medium pitched	Medium Pitched	Medium Pitched	Low pitched	Low pitched	Medium pitched	Medium pitched
*Vizhi* (eye examination)	Normal	Normal	Normal	Normal	Normal	Normal	Normal	Normal
*Malam* (stool examination)	Normal	Normal	Normal	Normal	Normal	Normal	Normal	Normal

## 4 Diagnostic assessment

Patients were screened for Microalbuminuria first. Then, the albumin/creatinine ratio (ACR), Estimated Glomerular Filtration Rate (e GFR), routine urine examination, and diabetic profile, including HbA1c, were evaluated to confirm Diabetic Nephropathy. According to the Siddha system, the diagnosis is *Neerizhivinal erpatta Siruneeraga noi* (kidney disease induced as a complication of DM). Causes for proteinuria other than Diabetes, like other viral infections, multiple myeloma, and use of non-steroidal anti-inflammatory drugs, non-diabetic nephropathies, and glomerulopathies were ruled out by urine routine and microscopic examination, complete blood count, and renal ultrasound scan done to exclude the obstructive renal disease and to assess renal anatomy and size [16].

## 5 Therapeutic intervention

**Fig. 1:**
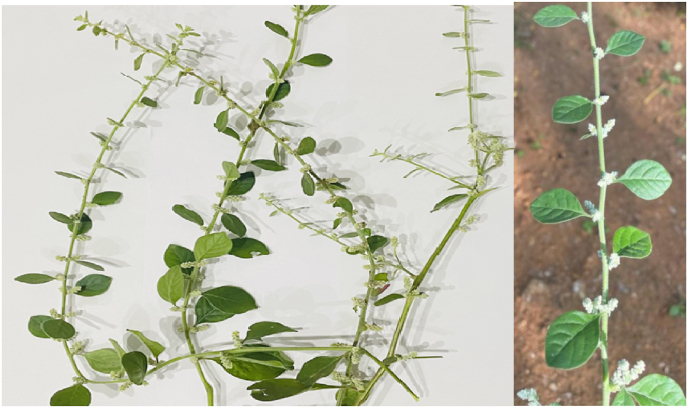
Sirupeelai (*Aerva lanata*) plant.

*Sirupeelai* (*Aerva lanata*) (Fig. 1) whole plants were collected from the local area/Medicinal Plants Garden; Botanical identity was authenticated, and whole plants were washed in pure water, cut into small pieces, and dried in sun shade. The dried plant is milled into the decoction powder of particle size passed through 40–60 mesh with a nominal mesh aperture of 5.6 mm. The powdered drug (*Sirupeelai Chooranam*- 6 gm) was boiled with water four times that of the drug (480ml) and made into ¼^th^ of it. The decoction in the dose of 120 ml was given twice daily before food. The drug was prepared daily just before administration due to the limited shelf life of the drug (3 hours). Sirupeelai Kudineer was administered twice daily for the first patient for five weeks. The second patient receives medication for three weeks; the third receives Sirupeelai kudineer for four weeks; and the fourth case receives it for two weeks. There were no Adverse Drug Reactions or Adverse events reported among the patients. Advised the patient to limit their intake of high-protein and high-carbohydrate foods. Avoid canned foods, fast food, processed meat, and sausages that are too salty. Use rock salt rather than table salt. Use fewer soft drinks, legumes, and milk products. Protein intake should be limited to 0.8g/kg body weight per day, and daily sodium intake should not exceed 1500 mg.

## 6 Follow-up and outcomes

The patient was advised to follow up once weekly for one month or until the parameters get normal. Assessment is considered in subjective and objective parameters. Symptomatic improvement or total cure of symptoms in patients, a significant reduction in the level of blood urea, serum creatinine, urine microalbumin, ACR or improvement in e GFR value, delayed progress of chronic kidney disease, and Improvement in Quality-of-life measures (QOL) was considered as a successful outcome of the treatment.

In this case series, all four men were in the sixth decade of life. The renal profile trends of the patients during medication are summarized in Table 1. The eight kinds of examination techniques used in the Siddha system for the diagnosis and prognosis of the disease (*Enn vagai thervukal*) are given in Table 2. Finally, the assessment of the CKD QOL questionnaire's positive findings before and after treatment is summarized in Supplementary Tables 1 and 2. Remarkable reduction in blood urea and serum creatinine occurred in all the cases after medication. ACR value and urine creatinine level was decreased for two cases, but both parameters were increased after treatment for the other two patients. Urine microalbumin was elevated in two cases after taking medicine and reduced in the other two cases. GFR value increased in three instances but mildly reduced in one after medication. Urea creatinine ratio reduced in all after treatment. However, an increase in blood sugar levels was noted (Blood parameters levels before, during, and after treatment are given in Table 1). Symptomatic improvement was observed in all the patients. The severity of the subjective parameters, such as bilateral pedal edema, General weakness, itching, excessive thirst, joint pain, frequent nocturnal micturition, and muscle cramps, were significantly reduced during medication. In most cases, *Neikkuri* (Siddha urine examination) showed (Supplementary Fig. 1) the transformation from *Kapha* to *vatha* and *pitha* humor and from slowly spreading to fast-spreading nature (details are listed in Table 2). The assessment of CKD QOL also showed significant improvement in major symptoms after treatment and improved quality of life.

## 7 Discussion

In this case series, we presented the case reports of four patients of diabetic Nephropathy who were treated with the monoherbal siddha formulation *Sirupeelai kudineer.* According to Siddha literature, Neerizhivu noi is a condition due to vitiated *Iyya* (One of the three types of the bodily composition according to Siddha concept) humor primarily affecting the seven body constituents – *Udal thadhukkal* (*Saaram* or nourishing juice, *Cheneer* or blood, *Oon* or Muscles, *Kozhuppu* or Fat, *Enbu* or bone, *Moolai* or marrow, *Sukilam*/*Sronitham* or Semen/Ova) and the natural forces like *Keezhnokkukal* (excretory system) and then leading to emaciation and functional impairment [5]. The *Iyya* humor combines with deranged *Azhal* humor and causes generalized edema and the associated symptoms like dyspnoea, periorbital edema, skin dryness, malaise, giddiness, fainting, and vomiting. Finally, *Vali* humor combines with it and causes abdominal distension, abdominal pain, headache, chest pain, and malaise. Naadi of the patients also showed the predominance of pitha since pitha naadi is the main driving force in Madhumegam cases. The Siddha parameter *Neikkuri* (oil on urine sign) also shows a slowly spreading nature, especially after treatment, indicating a good prognosis as per siddha literature [18].

This study observed changes in hematological and biochemical parameters during and after the intake of Medicine. A gradual reduction in blood urea and serum creatinine was observed in the first case, and an increase in GFR was also noted, but there was an increase in Microalbumin and ACR during the treatment period. That may be due to glomerular hyperfiltration [19]. Case II showed a reduction in Microalbumin and ACR during the treatment period. Certain studies reported that albuminuria is a dynamic, fluctuating condition rather than a linearly progressive process [20]. In cases III and IV, there is a gradual reduction in blood urea and serum creatinine. Marked reduction in urine microalbumin and ACR also, but there is fluctuation in e GFR values. Blood urea level was significantly reduced in all the cases. However, serum creatinine levels mildly increased in three cases somewhere during the treatment period but decreased in all the patients after treatment. Marked reduction in blood urea and serum creatinine shows the nephroprotective action of the drug that has already been evident from pharmacological studies [21]. The estimated GFR also improved in three cases after treatment but mildly reduced in one patient.

Symptomatic improvement was also observed in all the patients. However, the blood sugar levels of the patients did not reduce much during the treatment. That may be due to the sudden stoppage of some oral hypoglycaemic drugs the patient was taking. Since we intended to assess the efficacy of *Sirupeelai Kudineer* for a short duration (while taking Sirupeelai Kudineer for the above-mentioned period of two to five weeks), we stopped all other siddha internal medicines and some allopathic medicines the patients were already taking. It was stopped mainly to avoid other drug interactions, and the patient also wants to reduce those medicines. No adverse events or side effects were noted. Since Sirupeelai is also proven to have antidiabetic activity, we gave that for the prescribed period. However, some patients’ blood sugar levels are not much improved, so we advised some other antidiabetic medicines later.

This series of four cases showed improvement in CKD QOL and some biochemical parameters. It would help establish new hope in treating Diabetic Nephropathy, which usually progresses to end-stage renal disease. However, in some instances, more studies are needed to determine the reason for the fluctuation in the levels of Urine Microalbumin and GFR values, thereby proving the scientific facts. In this Diabetic Nephropathy treatment with the *Sirupeelai Kudineer*, no adverse effects were observed during and after the study. Hence this drug *Sirupeelai Kudineer* may be used as supportive therapy in Patients with Diabetic Nephropathy along with other treatment methods.

## 8 Conclusion

In this case series, Considering the patient's symptomatic improvement and improvement in specific renal parameters, it is being concluded that the drug *Sirupeelai Kudineer* may benefit those patients with diabetic Nephropathy if the glycemic control is maintained in the patient. Moreover, in Diabetic Nephropathy patients with stable vital signs, the drug *Sirupeelai Kudineer* can be used as adjuvant therapy in addition to the other allopathic medications. On the other hand, the study has created the pathway for further studies in managing Diabetic Nephropathy.

## Patient perspective

The patients self-reported that they were contented with the treatment as they had a considerable reduction in symptoms experienced, and their quality of life was improved after the medication.

## Informed consent

Written informed consent was obtained from all the cases.

## Author contributions

Author 1 (PP): Investigation, analysis, data curation, drafting, revising, final approval, and accountability for all aspects of the work. Author 2 (GSL): Conception, design, acquisition, investigation, supervision, analysis, interpretation, drafting, revising, final approval, and accountability for all aspects of the work. Author 3 (AS): Analysis, interpretation, drafting, revising, final approval, and accountability for all aspects of the work. Author 4 (AK): Supervision, final approval, and accountability for all aspects of the work.

## Funding sources

Central Council for Research in Siddha, Ministry of AYUSH, Government of India.

## Declaration of generative AI in scientific writing

None.

## Conflict of interest

None.
