# Supplementary figures and images for: Treatment of early-stage diabetic nephropathy with Siddha drug Sirupeelai Kudineer: A case series
Source: J Ayurveda Integr Med. 2024 Dec 2;15(6):100993. doi: 10.1016/j.jaim.2024.100993 (PMC11652741; doi:10.1016/j.jaim.2024.100993)

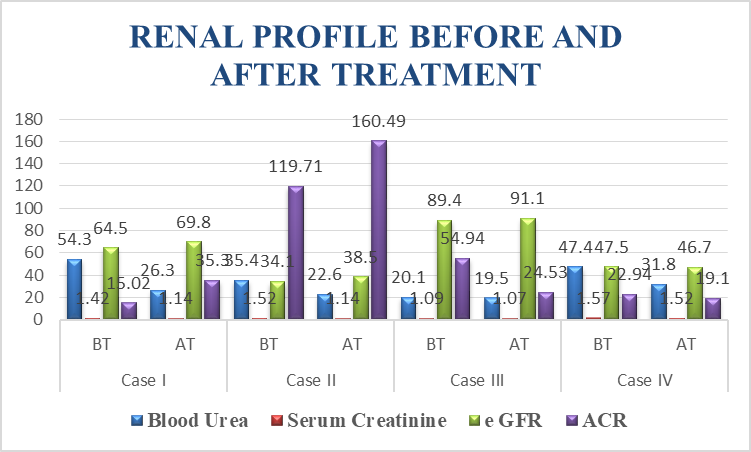

Supplement: Multimedia component 1 [file mmc1.doc]
