# Supplementary material for: Treatment of early-stage diabetic nephropathy with Siddha drug Sirupeelai Kudineer: A case series
Source: J Ayurveda Integr Med. 2024 Dec 2;15(6):100993. doi: 10.1016/j.jaim.2024.100993 (PMC11652741; doi:10.1016/j.jaim.2024.100993)
Supplement: Multimedia component 2 [file mmc2.docx]

**NEERKKURI & NEIKKURI PICTURES OF THE PATIENTS**

| **Case I - Before Treatment** | | | |
| --- | --- | --- | --- |
| 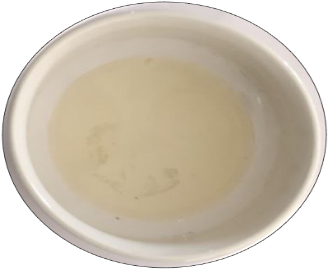 | 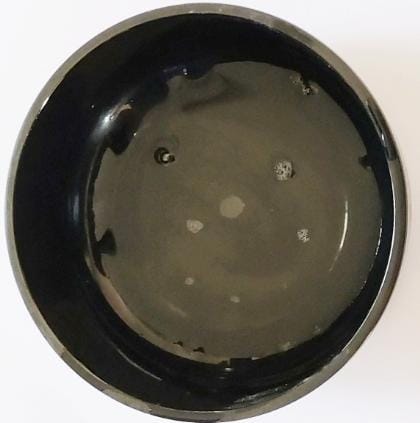 | **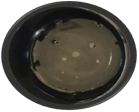** | **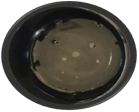** |
| Neerkkuri | Neikkuri | | |
|  | At the moment | 3 Minutes | 10 Minutes |
| Straw-colored, pearl Shaped, not spreading- Kapha neer | | | |
| **Case I - After Treatment** | | | |
| 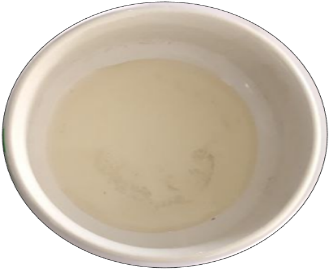 | 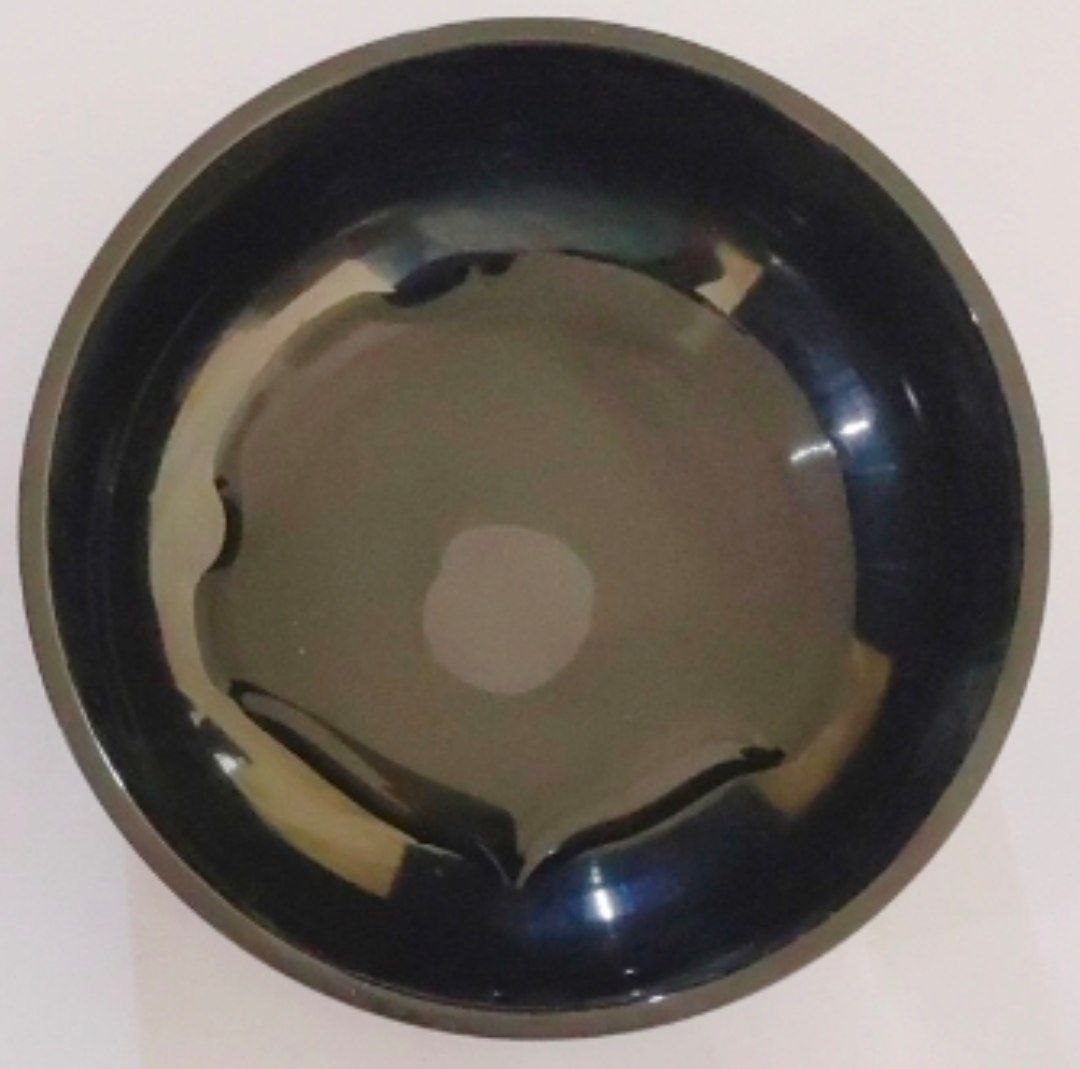 | 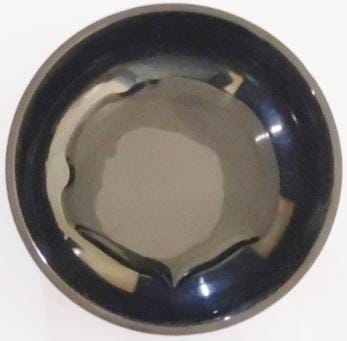 | 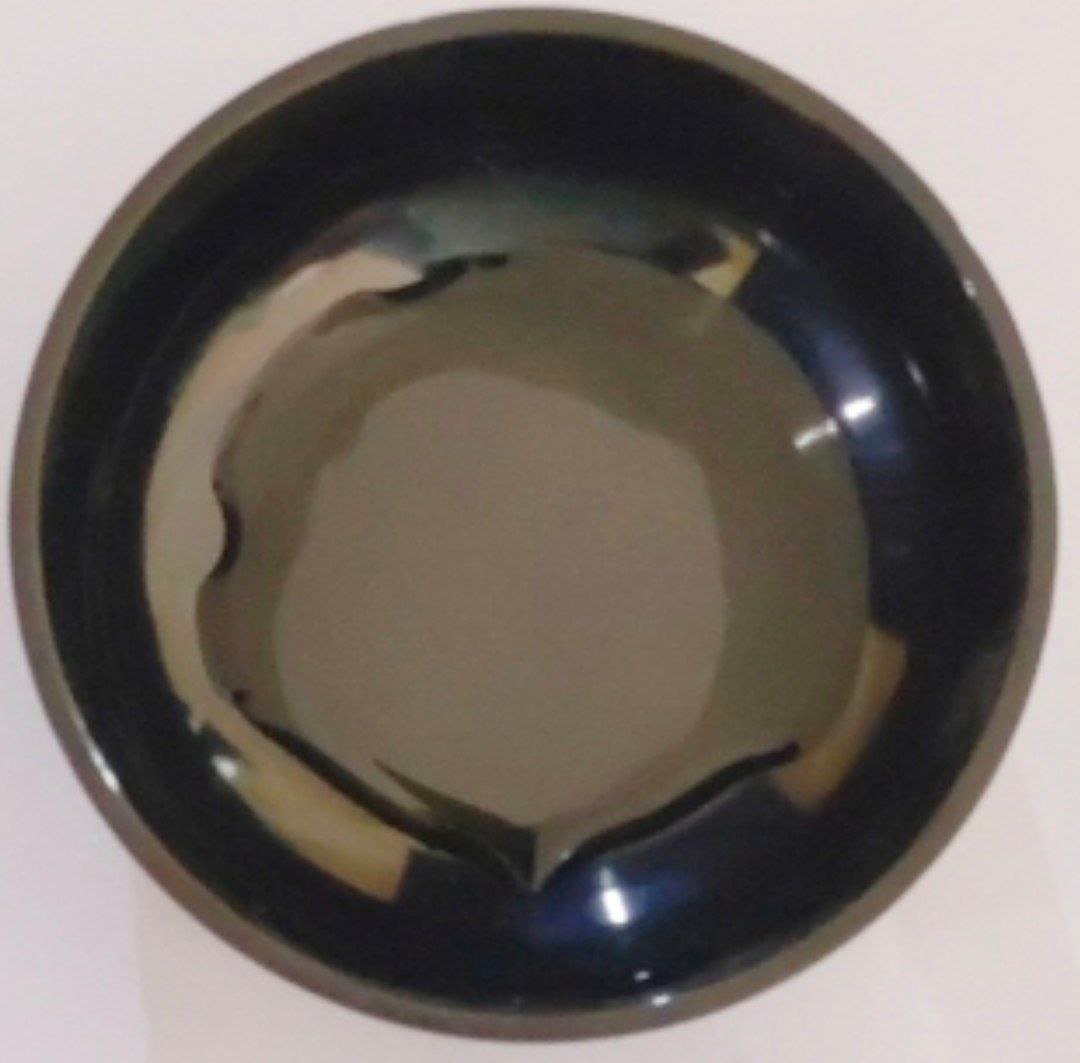 |
| Neerkkuri | Neikkuri | | |
|  | At the moment | 3 Minutes | 10 Minutes |
| Straw colored, round pattern, Steady spread – Pitha neer | | | |
| **Case II - Before Treatment** | | | |
| 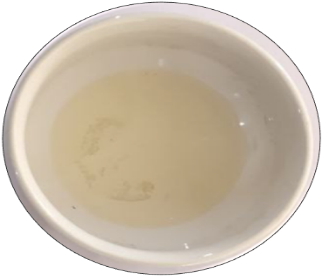 | 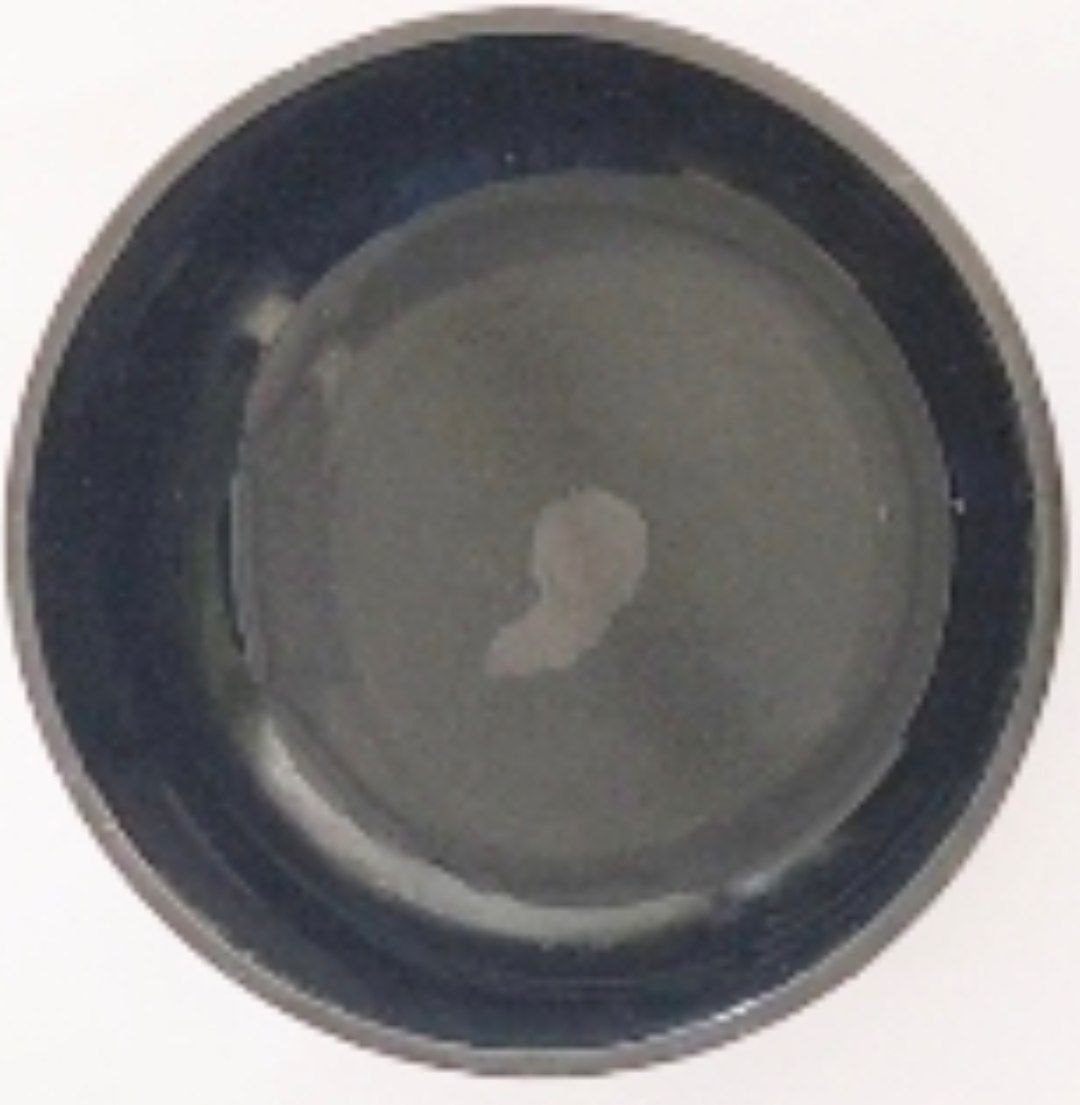 | 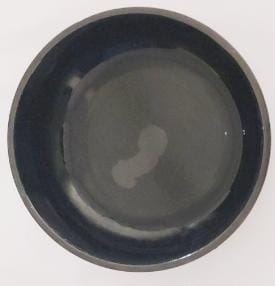 | 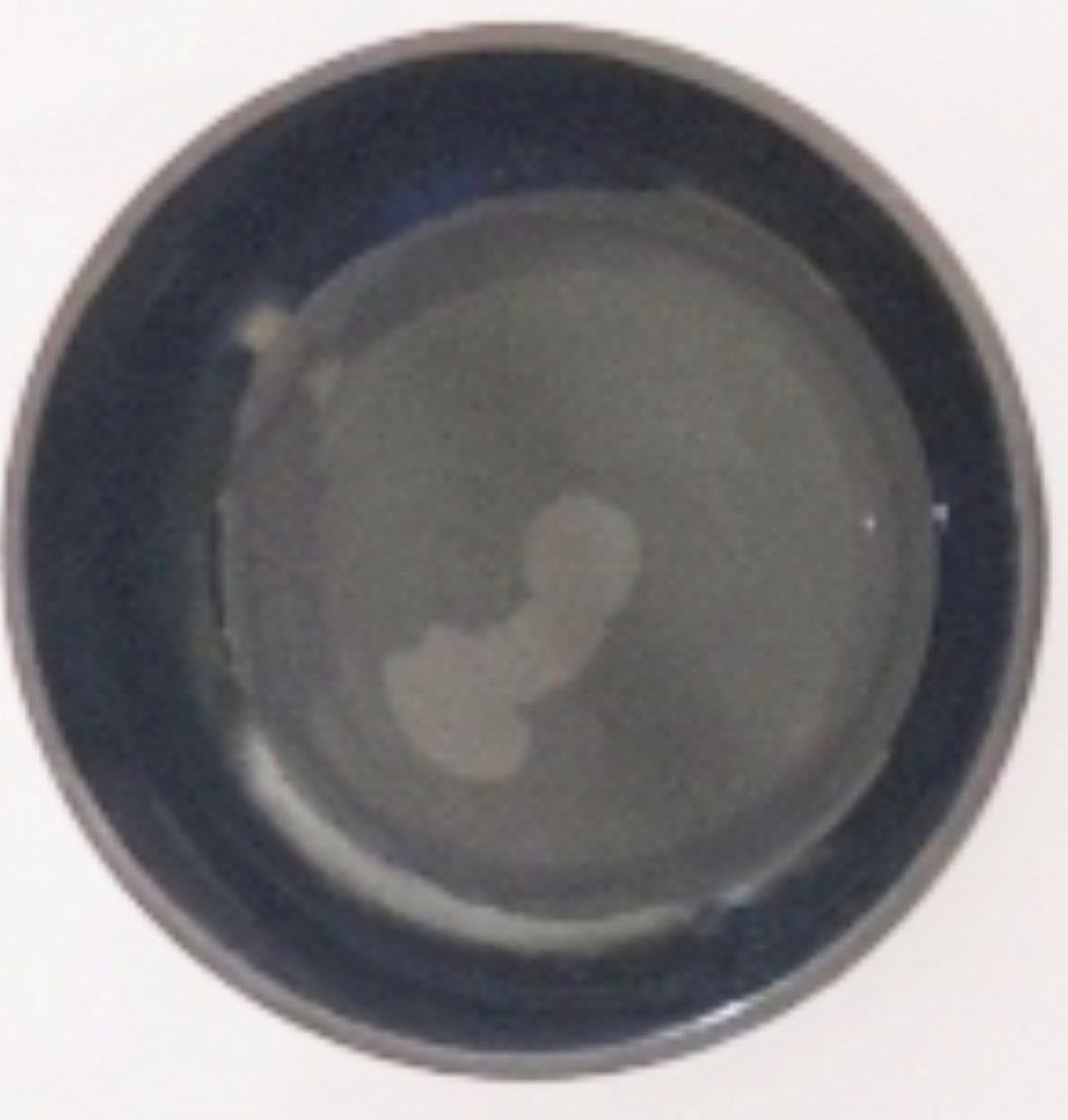 |
| Neerkkuri | Neikkuri | | |
|  | At the moment | 3 Minutes | 10 Minutes |
| Yellowish, linearly spreads – Vatha neer | | | |
| **Case II - After Treatment** | | | |
| 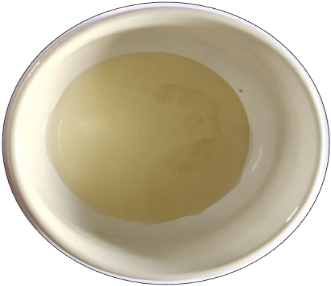 | 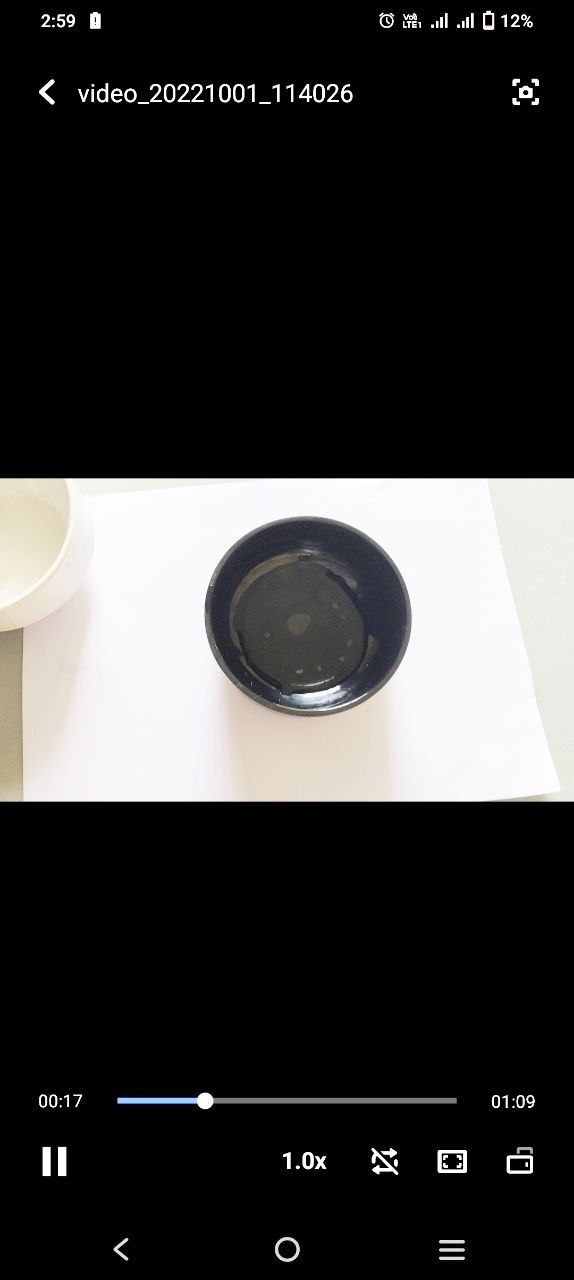 | 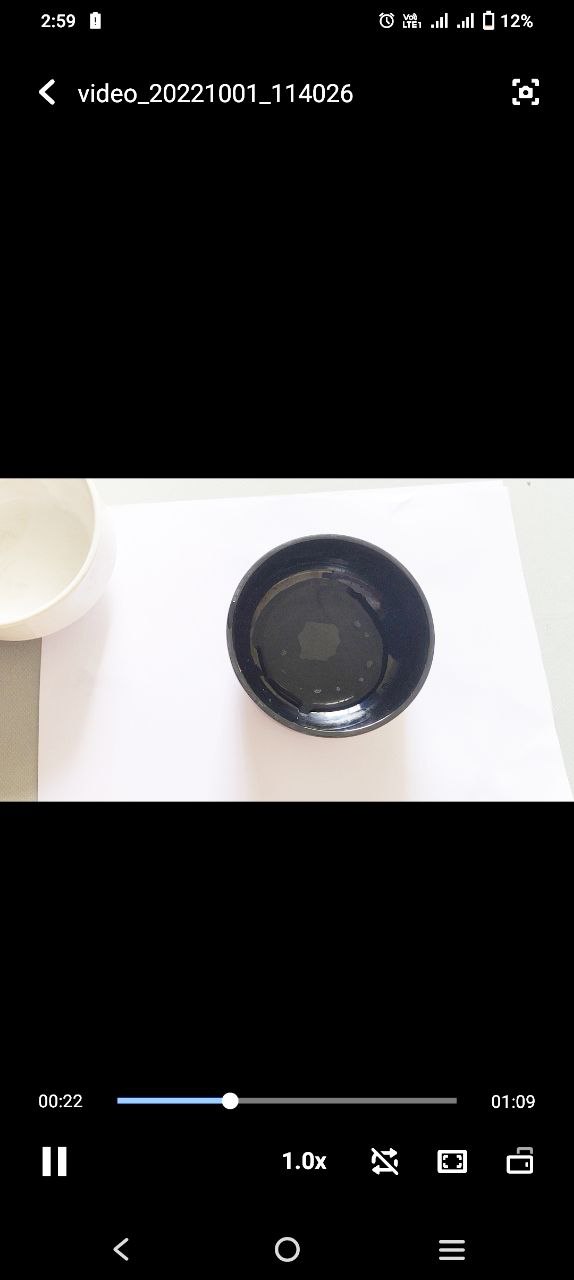 | 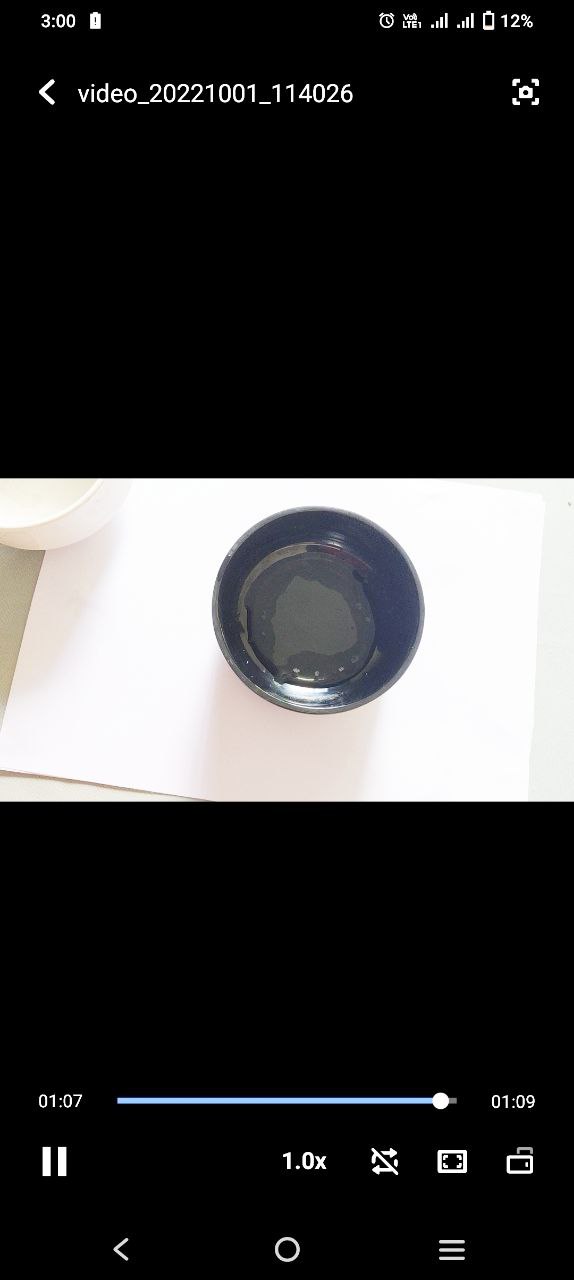 |
| Neerkkuri | Neikkuri | | |
|  | At the moment | 3 Minutes | 10 Minutes |
| Yellowish, linear initially, then spread all over to sides – Vatha pitha neer | | | |
| **Case III - Before Treatment** | | | |
| 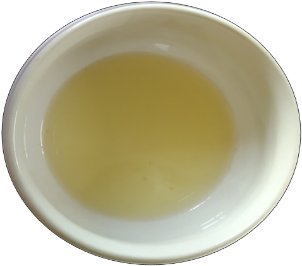 | **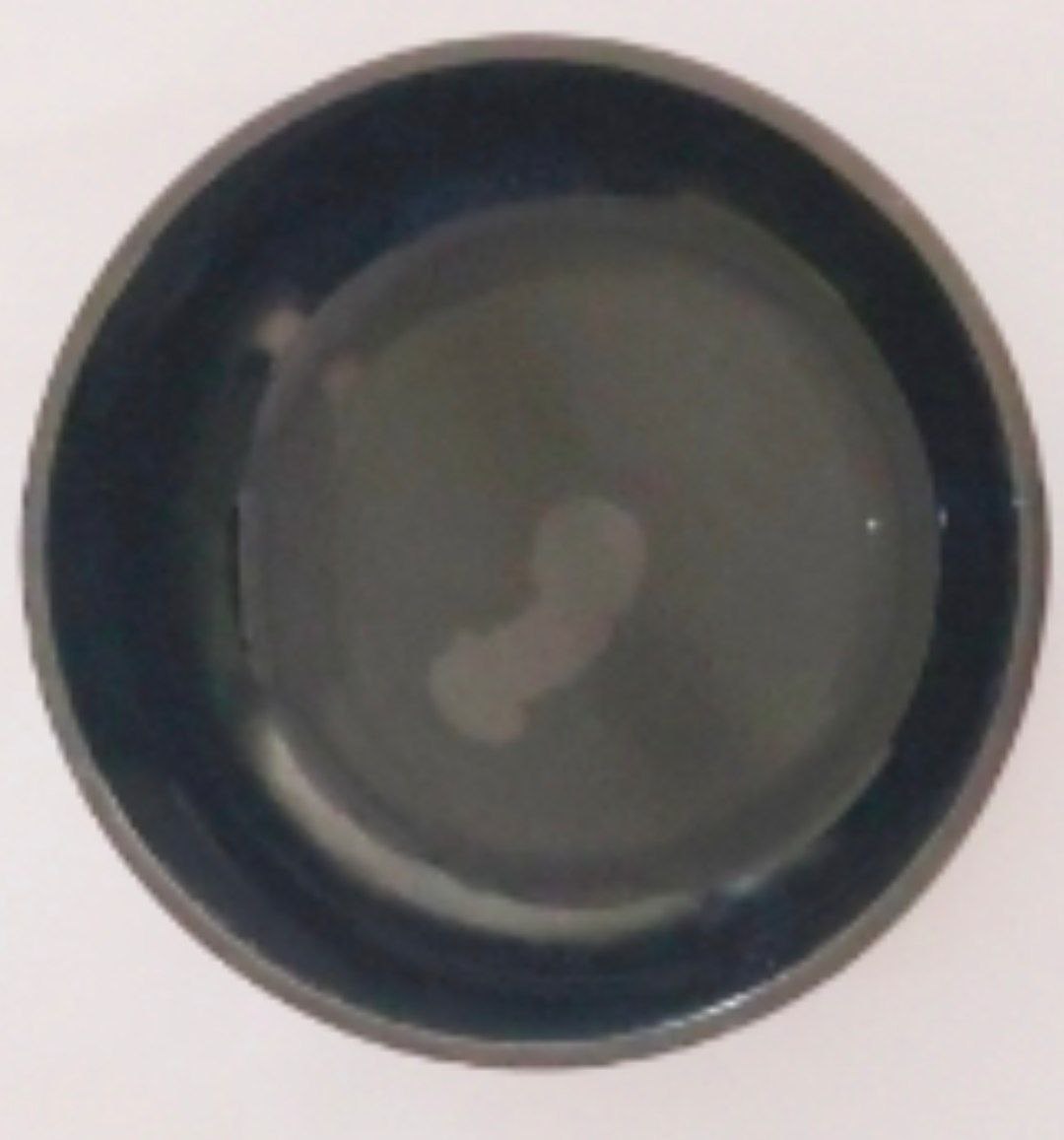** | 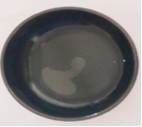 | **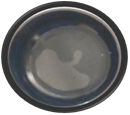** |
| Neerkkuri | Neikkuri | | |
|  | At the moment | 3 Minutes | 10 Minutes |
| Straw colored, kidney-shaped- Vatha pitha neer | | | |
| **Case III - After Treatment** | | | |
| 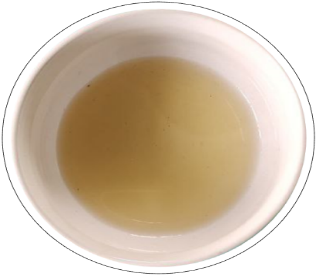 | 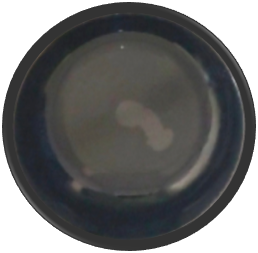 | 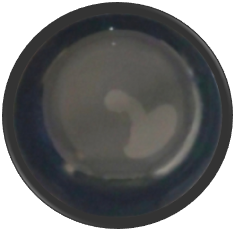 | 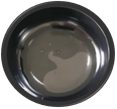 |
| Neerkkuri | Neikkuri | | |
|  | At the moment | 3 Minutes | 10 Minutes |
| Straw colored, kidney-shaped- Vatha pitha neer | | | |
| **Case IV** **- Before Treatment** | | | |
| 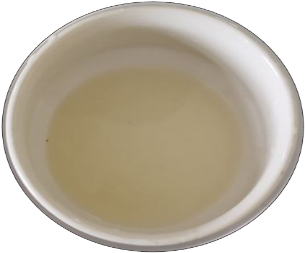 | 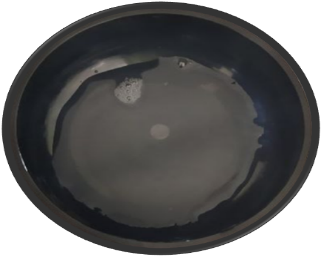 | **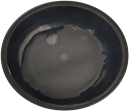** | **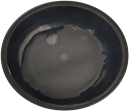** |
| Neerkkuri | Neikkuri | | |
|  | At the moment | 3 Minutes | 10 Minutes |
| Straw colored, pearl shaped – Kapha neer | | | |
| **Case IV - After Treatment** | | | |
| 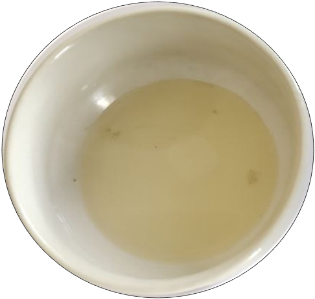 | 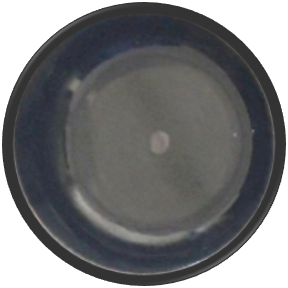 | 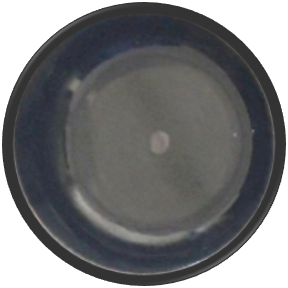 | 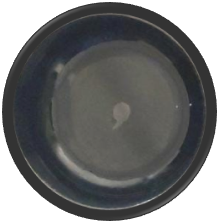 |
| Neerkkuri | Neikkuri | | |
|  | At the moment | 3 Minutes | 10 Minutes |
| Straw colored, parrot beak shaped – Kapha vatha neer | | | |

**Figure 2. Neerkkuri & Neikkuri Pictures of the Patients - Before and After Treatment.**
