# Supplementary material for: Treatment of early-stage diabetic nephropathy with Siddha drug Sirupeelai Kudineer: A case series
Source: J Ayurveda Integr Med. 2024 Dec 2;15(6):100993. doi: 10.1016/j.jaim.2024.100993 (PMC11652741; doi:10.1016/j.jaim.2024.100993)
Supplement: Multimedia component 3 [file mmc3.doc]

**Table 3: Assessment of CKD QOL Questionnaire - Gradation of Symptoms [17]**

| Gradation of Symptoms | |
| --- | --- |
| 1 | Extremely bothered |
| 2 | Very much bothered |
| 3 | Moderately bothered |
| 4 | Somewhat bothered |
| 5 | Not at all bothered |

**Table 4: Assessment of CKD QOL questionnaire – Before Treatment (BT) and After Treatment (AT)**

| **Clinical Features** | **Case I** | | **Case II** | | **Case III** | | **Case IV** | |
| --- | --- | --- | --- | --- | --- | --- | --- | --- |
|  | BT | AT | BT | AT | BT | AT | BT | AT |
| Fatigue/weakness | 5 | 5 | 5 | 5 | 2 | 4 | 2 | 3 |
| Dry skin | 5 | 5 | 5 | 5 | 4 | 4 | 2 | 3 |
| Itchy skin | 5 | 5 | 4 | 5 | 2 | 4 | 1 | 2 |
| Muscle soreness | 5 | 5 | 4 | 5 | 5 | 5 | 2 | 3 |
| Excessive thirst | 5 | 5 | 5 | 5 | 5 | 5 | 5 | 5 |
| Joint pain | 5 | 5 | 5 | 5 | 2 | 4 | 2 | 2 |
| Sleep during day | 5 | 5 | 4 | 4 | 4 | 5 | 4 | 5 |
| Cramps in muscles | 5 | 5 | 4 | 5 | 5 | 5 | 2 | 5 |
| Joint stiffness | 5 | 5 | 5 | 5 | 5 | 5 | 3 | 5 |
| Numbness in hands or feet | 3 | 4 | 2 | 2 | 5 | 5 | 2 | 2 |
| Bone aches | 5 | 5 | 5 | 5 | 3 | 4 | 2 | 2 |
| Lack of appetite | 5 | 5 | 5 | 5 | 2 | 5 | 5 | 5 |
| Trouble with memory | 2 | 2 | 5 | 3 | 3 | 3 | 3 | 3 |
| Shortness of breath | 2 | 3 | 5 | 5 | 5 | 5 | 1 | 2 |
| Swelling of ankles | 5 | 5 | 5 | 5 | 3 | 5 | 2 | 3 |
